# COVID-19 related psychosocial problems among university students in Mexico – a longitudinal qualitative examination

**DOI:** 10.3389/fpubh.2023.1160896

**Published:** 2023-06-14

**Authors:** Cecilia Martinez-Torteya, Caleb J. Figge, Laura I. Ramírez Hernández, Beatriz Treviño-de la Garza

**Affiliations:** ^1^Department of Psychiatry, University of Michigan, Ann Arbor, MI, United States; ^2^Department of Mental Health, John Hopkins Bloomberg School of Public Health, Baltimore, MD, United States; ^3^Department of Education, Universidad de Monterrey, San Pedro Garza García, Mexico; ^4^Department of Psychology, Universidad de Monterrey, San Pedro Garza García, Mexico

**Keywords:** COVID-19, Mexico, college students, qualitative, mental health

## Abstract

Research on the impact of the COVID-19 pandemic among college students around the world has primarily focused on their mental health symptoms and COVID-specific worry. However, contextually specific understanding of outbreak impacts is key to inform directed public health messaging and programming to improve wellbeing and coping. The current study aimed to identify the main psychosocial problems college students experienced during the first 6 months of the COVID-19 pandemic in Monterrey, Mexico. Participants were 606 college students (71% female) enrolled in a private university. Participants described COVID-related problems in an open-ended prompt as part of a longitudinal online survey: initially in May 2020, and then every 2 weeks for 3 months. Thematic analyses were conducted within a longitudinal inductive qualitative approach to rank responses by frequency across themes. Five major categories emerged. At baseline, over 75% of participants noted the outbreak negatively impacted their daily activities and responsibilities, 73% their mental health, 50% their physical health, 35% their interpersonal relationships, and 22% their economic situation. Concerns remained relatively stable throughout the follow-up period, with interpersonal and economic concerns becoming more prevalent as the pandemic progressed. Problems identified in this study can inform preventative measures for future health crises, including tailoring public health messaging and expanding access to contextually sensitive mental and behavioral health programming.

## 1. Introduction

The negative impacts of the COVID-19 pandemic in a wide range of domains of functioning, including physical and mental health, are well documented (1, 2). International studies reported increased stress and distress, sleep problems, posttraumatic stress symptoms, and emotional symptoms such as fear, confusion, irritability, frustration, anger, and depression in the initial stages of the pandemic (3–5). Importantly, those diagnosed or recovering from COVID-19, those with a previous psychiatric diagnosis, those who experienced longer duration of quarantine or financial loss, women, and young adults may be at higher risk for poor psychosocial outcomes (3, 6).

College students, specifically, show concerningly high levels of mental health problems across the globe (7, 8), with rates that significantly increased in response to the pandemic, confinement measures (4, 9), and widespread COVID-related worry (10). In addition to mental health symptoms, the pandemic has likely also impacted college students’ functioning more broadly: closure of schools, businesses, churches, and other community organizations can limit access to sources of income, social networks, support systems, and basic daily resources, leading to significant disruptions in their academic, social, and work activities (11–13). Therefore, understanding students’ perspective of the areas of their lives that were most impacted during the pandemic is key to informing prevention and intervention efforts and curtailing long-term negative outcomes.

In Mexico, research suggests college students experienced high levels of stress, anxiety and depression (14), COVID-related worry (15), and sleep difficulties (16) at the onset of the pandemic. However, understandings of the experiences and expressions of distress related to COVID-19, including the social ecological impact of psychosocial stress, academic challenges, or financial loss, need to take into account the contextual and cultural environment through an *emic* framework (17). A large body of evidence points to culturally bound symptoms, likely not captured by Eurocentric mental health measures, as critical to understanding and treating problems in a given population and optimizing public health efforts (18, 19).

Contextually specific examinations of the psychosocial impact of the COVID-19 pandemic are critical for 1) informing directed and culturally sensitive public health messaging and directives, 2) identifying new or exacerbated psychosocial concerns that may persist over time, and 3) designing interventions that target a broad variety of pandemic-related psychosocial problems. The current study aims to explore COVID-related symptoms and problems among college students in Monterrey, Mexico between May and August 2020. We used free-list qualitative interviewing to elicit problems related to COVID-19 and the associated public health guidelines. We asked students to report the problems they experienced as a result of the pandemic every 2 weeks to assess potential changes in the types and perceived importance of difficulties. This study provides a contextually specific understanding of psychosocial concerns resulting from COVID-19 to contribute to the global literature in public mental health response. In addition, this study contributes a unique longitudinal qualitative methodological approach to measuring distress over time, as multiple quantitative studies have reported longitudinal changes in COVID-related symptomatology (20, 21). Results can inform public health policy and mental health programming with college students in Mexico and similar contexts to target pandemic-specific challenges.

## 2. Materials and methods

### 2.1. Participants and procedures

All procedures for this study were approved by the Institutional Review Board at Universidad de Monterrey. Participants were 606 college students enrolled in a private University in Monterrey, Mexico. Monterrey has over 5.13 million habitants (22) and is one of the main business and financial centers in the country, where more than 95% of the population has access to water, electricity, sewage and sanitation, and 88% report access to public or private health services (23).

Students included 434 females (71.6%), 167 males, and 5 non-binary students, ages 18 to 33 (M = 20.97, SD = 2.19). A range of monthly family-level income was reported, with 25.7% reporting less than 30,000 pesos (< ~$1,453 USD), 44% between 30,000 and 80,000 (~$1,453–3,875 USD), and 30.3% a family income greater than 80,000 (> ~ $3,875 USD); less than 10% of the sample reported a monthly household income under the poverty line (24). Participants represented all the academic Departments in the University; 22% were in their first year, 20% were in their second year; 20% were in their third year; 21% were in their fourth year; and 16% were in their fifth year or above. Most students (73%) reported living at home with their family, and 94% were living with their family during the pandemic. Also, 62% reported receiving financial aid or a scholarship to attend the university.

All students in the University were invited to participate in the study via an email sent by their Program Director and the social media networks of the College of Education and Humanities. Some instructors and student groups also shared information about the study in their courses or through social media. Interested students accessed a Qualtrics survey to confirm they met the recruitment criteria, completed informed consent, and then completed a 30-min survey about their demographics, mental health, adverse childhood experiences, social supports, and reactions to the COVID-19 pandemic and stay-at-home measures.

The study took place between May and August 2020. After the first case of COVID-19 was reported in Mexico on February 27th, 2020, cases steadily climbed, leading to a state of national emergency, declared on March 30th, that mandated the suspension of all non-essential economic, government, and social activities, including face-to-face classes. Despite continued increases in infection rates and mortality, the state of national emergency was lifted on June 1st 2020, and states developed guidelines for safely reopening governmental and economic activities, including mandatory face coverings, required social distancing, limited hours of operation and reduced capacity. However, all schools continued exclusively relying on online and distance programming. Despite these measures, cases continued to increase exponentially during the summer, with 606,036 infections and 65,241 deaths reported in the country by September 1st, 2020 (25).

The baseline survey was available from May 13 to May 27, 2020. Then, all participating students were invited via email every 2 weeks to complete brief surveys for the following 14 weeks (June to August); 520 students completed at least one of the summer surveys, and completion rates were as follows: T2 = 478, T3 = 399, T4 = 386, T5 = 352, T6 = 310, T7 = 296, T8 = 279. All recruitment and data collection procedures were completed online as all in-person activities in the University were suspended since March 2020.

### 2.2. Qualitative approach

An inductive approach was used to collect and analyze the data (26). Inductive methods allow for a data-driven derivation of findings guided by study aims and research questions. The rapid qualitative data collection and analysis approach was adapted from the Design, Implementation, Monitoring, and Evaluation of mental health and psychosocial assistance programs for trauma survivors in low resource countries – Module 1 (27). The DIME procedure is designed to explore expressions of distress to inform assessment and treatment design in global settings. For the current study, we examined responses from the open-ended question:

*What are the three main problems you have faced due to COVID-19 and the stay-at-home measures? You can include problems related to your health (headache, stomachache, fatigue, insomnia*, etc.*), mental health (distress, stress, sadness, mood changes, etc.), interpersonal (arguments with family, partner, friends, loneliness*, etc.*), academic (low concentration, low motivation, low achievement, too much load), work-related (loss of a job, difficulties with home office*, etc.*), financial (less income, business closure*, etc.), *or other situations you are experiencing. Describe each one concretely (in 1 to 5 words)*.

Survey questions were administered in Spanish, the primary language spoken by participants. Participants were prompted to provide three COVID-related problems in open free text response boxes. Students answered the initial survey between May 13 and May 27, 2020, about 8 weeks after the initial suspension of in-person classes and non-essential activities took place in Mexico, in March. Then, they received an invitation to answer the same question in regards to the most significant problems experienced during the past 2 weeks throughout June, July and August (T2 to T8).

The full research team included eight Mexican research staff and one American global mental health researcher. The American investigator worked closely with the Mexican research team throughout project development, implementation, and dissemination.

### 2.3. Data coding

Two of the authors (CMT, CF) and five undergraduate research assistants underwent the following procedures to create the final codebook: (1) Coders independently reviewed responses to the initial survey from 100 participants, (2) One hundred participant’s responses (total responses = 331) were consensus coded, (3) coders met to review codes, reconcile discrepancies, and create a preliminary codebook, (4) 50 additional participant responses were consensus coded according to the preliminary codebook, (5) coders met to reconcile discrepancies and create a final codebook, and (6) 75 participant responses were consensus coded using the final codebook, from which percent agreement and Kappa were calculated with the master coder (CMT) and with each other. All five coders had above 90% agreement with the master coder (*M* = 91.69%) and with each other (*M* = 93.31%) and above.90 Kappa with the master coder (*M* = 0.91) and with each other (*M* = 0.93). These steps were taken to support reliability and validity (28). Split coding was used to code all timepoints using the final codebook. Additional consensus meetings were held regularly to discuss cases that were difficult to code.

Codebook development and refinement resulted in 5 theme categories, with 23 themes and 9 subthemes. See Table 1 for an outline of codebook themes.

**Table 1:** Codebook structure and themes.

Category	Theme	Subtheme
1. Mental Health	1.1 Emotional problems	1.1a Sadness or depression
		1.1b Anxiety or worry
		1.1c Anger or frustration
	1.2 Cognitive problems	
	1.3 Behavior problems	
	1.4 Stress	
	1.5 Self-esteem	
2. Physical Health	2.1 Pain	2.1a Headache
		2.1b Stomach ache
	2.2 Physical tiredness	
	2.3 Sleep problems	
	2.4 Physical activity	
	2.5 Eating	
3. Daily Activities and Responsibilites	3.1 Concentration problems	3.1a Academic concentration
	3.2 Poor motivation	3.2a Poor academic motivation
	3.3 Low achievement	
	3.4 Too much responsibility	
	3.5 Academic problems	3.5a Too much academic work
	3.6 Work problems	3.6a Loss or difficulty finding work
4. Interpersonal Relationships	4.1 Isolation or loneliness	
	4.2 Relationships with friends or colleagues	
	4.3 Family relationships	
	4.4 Partner relationship	
5. Economic	5.1 Family economic problems	
	5.2 Individual economic problems	

Each participant response was assigned a unique code (i.e., no single response was assigned multiple codes). In the case of a participant listing 2 or more distinct responses in a single text box, responses were split and analyzed as separate entries. Responses were aggregated for each code and within categories, both by a proportion of total responses and by total number of participants.

## 3. Results

Only 4% of the sample reported having contracted COVID-19 at some point during the study, but 60% had a loved one who tested positive. Participants reported a wide range of domains impacted by the COVID-19 outbreak and associated restrictions/guidelines. In the initial survey, over 75% of participants (*n* = 456) noted the outbreak and restrictions negatively impacted their daily activities and responsibilities (*actividades y responsabilidades diarias*), and about 73% (*n* = 443) reported impact on their mental health (*salud mental*). Over 50% (*n* = 312) of participants noted COVID negatively impacted their physical health (*salud física*), 35% (*n* = 215) their interpersonal relationships (*relaciones interpersonales*), and 22% (*n* = 133) their economic (*económico*) situation. Please see Table 2 for reporting by category, ranked by frequency. Longitudinally, concerns related to daily activities and responsibilities showed the most variability, becoming less prevalent over time. Problems with daily activities decreased from 75% at T1, to 66% at T2, and 55% at T3, reaching its lowest point at T8. This means only about 47% of students mentioned daily activities as one of their most significant difficulties by the end of August. On the other hand, reporting of interpersonal and economic problems became more common as the pandemic progressed. About 29% of students reported economic difficulties the summer months (T3 and T4), and 45% of students were concerned about their relationships at T2, T3, and T5. Notably, mental health was the top concern for students throughout the summer, with close to 70% of students reporting this concern most weeks. Physical health concerns showed some variation throughout the summer months, with a low frequency of 35% at T2 and a high frequency of 46% at T6.

**Table 2:** Theme responses, ranked by frequency as a percentage of participants.

Category/% of participants	T1	T2	T3	T4	T5	T6	T7	T8
Daily activities and responsibilities	75.25	68.2	54.6	57.1	50.9	47.7	50.7	47.3
Mental health	73.10	71.3	66.9	67.5	66.5	72.6	67.9	69.5
Physical health	51.49	34.9	40.1	41.8	38.6	43.2	45.6	45.2
Interpersonal relationships	35.48	45.0	44.9	36.9	44.6	40.3	42.2	35
Economic	21.95	28.2	29.1	29.1	27.6	25.5	23.6	26.9

**Figure 1:**
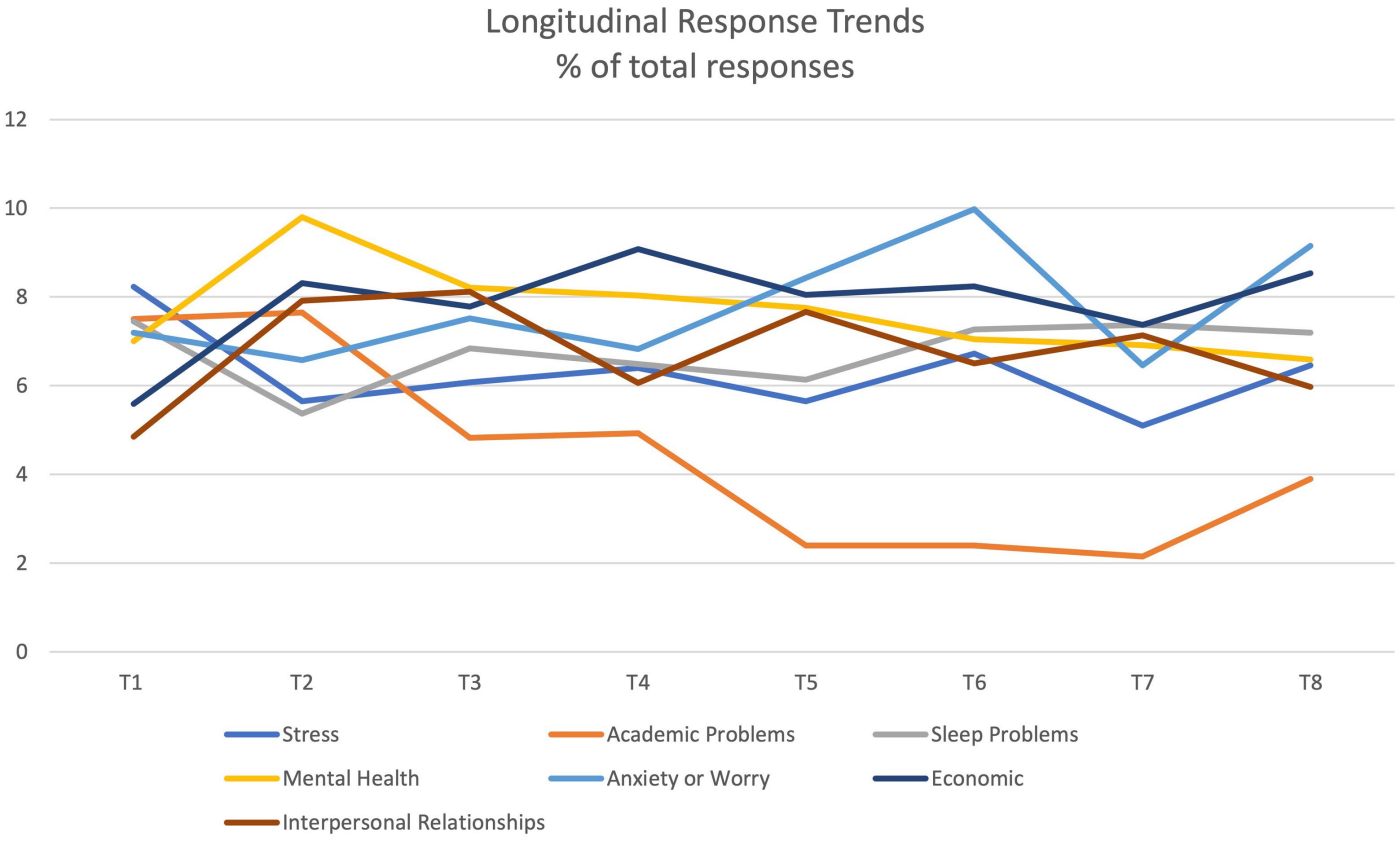
Subtheme longitudinal response trends (*N* = 2041).

At subtheme level, we evaluated each subtheme as a proportion of total responses (*N* = 2,041). The most frequently reported areas impacted by the outbreak and associated restrictions at T1 were stress (*estrés*, 8.23%), academic problems (*problemas académicos*, 7.5%), problems with sleep (*problemas de sueño*, 7.45%), anxiety or worry (*ansiedad o preocupación*, 7.2%), and mental health problems generally (*salud mental*, 7.01%; see Table 3). Over time, academic problems became less frequently mentioned as a concern (only 2.15% of responses at T7, see Figure 1), but problems with sleep, mental health, and anxiety or worry continued to be one of the five most commonly reported difficulties most weeks. In addition, economic problems became one of the two most commonly reported problems in a large proportion of the summer time points (T4-T8), while interpersonal relationships were also one of the top five concerns throughout the summer.

**Table 3:** Most frequent subthemes, ranked by frequency as percentage of responses (*N* = 2,041).

Category/ % of total codes	T1	T2	T3	T4	T5	T6	T7	T8
Stress	8.23%^1^	5.645%	6.07%	6.40%	5.65%	6.72%^5^	5.10%	6.46%^5^
Academic problems	7.50%^2^	7.65%^4^	4.83%	4.93%	2.39%	2.39%	2.15%	3.90%
Sleep problems	7.45%^3^	5.37%	6.84%^5^	6.49%^5^	6.13%	7.27%^3^	7.37%^2^	7.20%^3^
Mental health	7.01%^4^	9.80%^1^	8.21%^1^	8.04%^2^	7.76%^3^	7.05%^4^	6.92%^4^	6.59%^4^
Anxiety or worry	7.20%^5^	6.58%^5^	7.52%^4^	6.83%^3^	8.43%^1^	9.98%^1^	6.46%^5^	9.15%^1^
Concentration problems	5.73%	3.49%	3.25%	3.46%	3.35%	3.25%	2.61%	3.29%
Economic	5.59%	8.32%^2^	7.78%^3^	9.08%^1^	8.05%^2^	8.24%^2^	7.37%^1^	8.54%^2^
Interpersonal relationships	4.85%	7.92%^3^	8.12%^2^	6.06%	7.66%^4^	6.51%	7.14%^3^	5.98%
Physical health	4.02%	5.10%	6.50%	6.83%^4^	6.32%^5^	6.29%	6.01%	6.46%^5^

Superscript numbers indicate the 5 most common concerns for that week.

## 4. Discussion

Shortly after the beginning of the COVID-19 pandemic, during national lockdown conditions, about three quarters of our sample of university students in Monterrey, Mexico reported experiencing difficulties with their daily activities and responsibilities (including academic and professional activities) or their mental health functioning. Over half reported an increase in physical health problems due to the outbreak and restrictions. In addition, about one-third reported problems with interpersonal relationships and one-quarter faced economic problems due to COVID-19. This is consistent with findings of challenges associated with COVID-19 and related public health guidelines and restrictions, including stress, worry, economic challenges, and disrupted academic, occupational, and interpersonal functioning (7, 29, 30). Our study adds to previously published qualitative findings of disruption in personal, academic, and professional plans, emotional problems, loneliness and isolation among a small sample from a public university (31). Our findings extend previous research through the use of qualitative data, underscoring public health intervention efforts need to target multiple areas of functioning among young adults, as difficulties cut across multiple contexts, and impairments likely cascade from one domain to another (e.g., financial difficulties leading to higher levels of stress, and higher levels of stress increasing physical symptoms).

As the pandemic progressed, students continued reporting mental health difficulties, disruptions to daily activities and responsibilities, and physical health problems as their most common concerns. However, we observed slight longitudinal changes in the most salient problems identified. Over time, students expressed less frequent difficulties with their daily activities and responsibilities. This may reflect resilience and adaptation to a “new normal,” which is consistent with frameworks of individual and community adaptation to crisis and disaster (32). For example, a meta-analysis found that the negative psychological effects documented immediately after pandemic onset gradually decreased to baseline levels about 2 to 4 months later (33). However, findings may also be related to the timing of our data collection: many students were not enrolled in courses during the summer, and those enrolled had a smaller academic load as compared to the Winter semester. As such, students may have experienced significantly fewer academic difficulties, one of the most frequently mentioned subthemes of the daily functioning domain.

On the other hand, physical health problems became more prevalent over time. Somatization symptoms during the COVID-19 pandemic have similarly been reported among University students internationally (34, 35). The increase in health problems over time may reflect a pandemic “allostatic load,” or the bodily wear and tear associated with prolonged exposure to stressors (36). Notably, physical health has wide-ranging impacts in academic functioning (37), psychological well-being (38), and general quality of life (39). Longitudinal changes in prevalence observed in this study provide a unique contribution to the public health crisis literature, suggesting crisis response policy should be sensitive and responsive to both short- and long-term psychosocial response patterns.

Among subtheme level responses, stress, academic problems, sleep problems, mental health difficulties, and anxiety/worry were among the most frequently reported concerns at baseline. Notably, the most frequently reported problems align with a cluster of symptoms that are characteristic of a fear and anxiety-based response, including worry, disrupted sleep, difficulty concentrating, and stress. This response may help characterize the initial reactions to the COVID-19 outbreak, as the initial data collection was relatively shortly after the outbreak (about 2 months following the first cases in Monterrey). Our results are similar to a handful of other studies examining initial responses to the COVID-19 outbreak in Mexico and Latin America. For example, an online survey of a community sample in Mexico found 21% of participants reported severe symptoms of anxiety and 28% reported severe symptoms of depression in the early stages of the pandemic (40). In addition, a smaller online study reported about 29% of participants experienced high levels of anxiety, 17% experienced high levels of depression, and 33% considered COVID-19 related fear and worry interfered with their sleep (41).

As students continued reporting on their concerns throughout the summer, economic difficulties emerged as a highly pressing concern among students in our sample. This is noteworthy, considering only about 10% of the sample fell below the federal poverty line at T1. The economic impacts of the pandemic are well-documented. Research in the US reports almost half of all students in their sample lost their employment or their hours were reduced (42). Economic difficulties can also trigger worry and insecurity about the future, and hinder access to the resources needed for academic success [space, access to a computer or internet, etc., (43, 44)].

Our findings can also be interpreted in light of previous research of Mexican college students’ responses to previous health emergencies, such as the 2009 A/H1N1 Influenza. Similar to our findings, Infante et al. (45) identified that university students felt worried and anxious at the beginning of the influenza epidemic, but they observed that, after long periods of isolation depression became more prevalent. Relatedly, Estrada (46) reported that stay at home orders were associated with tension within the family environment due to increased interaction time. Taken together, these studies provide additional support to the need for multilevel public health intervention that addresses mental health in the context of pandemic-related economic, interpersonal, and physical health challenges.

### 4.1. Strengths and limitations of this study

To our knowledge, this is one of the first longitudinal studies of the psychosocial impact of COVID-19 among college students in Mexico. By using an open-ended longitudinal qualitative design, this study captures participant-generated problems over time that are not bound by items on existing measures, reducing risk of researcher bias, “category truncation,” and eliminating the need to implement multiple measures specific to areas of functioning. Findings further support the use of open-ended symptom assessments to capture salient problems not captured by existing measurement tools (18, 19). This approach allows for participant-driven responses that allow future public health crisis responses to be tailored and adaptive to contextual and longitudinal psychosocial needs.

Current findings indicate university students in this sample associate a range of psychosocial, academic, health functioning, and economic problems with the COVID-19 outbreak and associated restrictions. Problems described here should inform future pandemic response in this region and elsewhere, including public health messaging, community-level preventative programming and psychosocial assessment as a part of infectious disease-related assessment and care. It is clear from the current data and other emerging findings that a focus on mental health, physical health, and social connections and resources are a critical for facilitating cross-domain recovery from pandemic-related impacts.

Despite its longitudinal design, the current study assessed Mexican college students during the first few months of the pandemic, during a period where Universities were closed and vaccines were not available. Additional qualitative research is needed to better understand the evolving concerns and needs of university students, as they navigate the return to in-person classes. In addition, men and commonly marginalized populations (LGBTQ, ethnic minority, low-income students) were under-represented in our sample; future studies may benefit from exploring unique concerns of these populations.

## 5. Conclusion

The COVID-19 outbreak and the accompanying guidelines, restrictions, and closures impact a wide range of psychosocial, physical health, academic, and economic domains. In this sample of university students in Monterrey, Mexico, a majority noted problems with mental health, physical health, and daily activities and responsibilities due to COVID-19. Stress, mental health symptoms, anxiety, worry, and challenges with academics, sleep, and concentration were among the most frequently reported problems in this sample. It is critical to screen for problems and symptoms specific to the context and population to accurately and effectively assess, treat, and monitor the impact of public health emergencies and improve wellbeing and resilience. In addition, problems identified in this study can inform preventative measures for future public health crises, including tailoring guidelines and other messaging, disseminating information, and expanding access to mental health care early in the outbreak response.

## Data availability statement

The raw data supporting the conclusions of this article will be made available by the authors, without undue reservation.

## Ethics statement

The studies involving human participants were reviewed and approved by Comité de Ética en Investigación – Escuela de Medicina de la Universidad de Monterrey. The patients/participants provided their written informed consent to participate in this study.

## Author contributions

CM-T, CF, LRH, and BT-d contributed to the preparation of this manuscript. CM-T, CF, and LRH contributed to conceptualization and study design, CF and CM-T contributed to data coding, analyses, and manuscript writing. BT-d contributed to data collection and manuscript writing. All authors contributed to the article and approved the submitted version.

## Funding

This research was supported by a grant from Universidad de Monterrey (UIN #20538).

## Conflict of interest

The authors declare that the research was conducted in the absence of any commercial or financial relationships that could be construed as a potential conflict of interest.

## Publisher’s note

All claims expressed in this article are solely those of the authors and do not necessarily represent those of their affiliated organizations, or those of the publisher, the editors and the reviewers. Any product that may be evaluated in this article, or claim that may be made by its manufacturer, is not guaranteed or endorsed by the publisher.

## References

[1] Jin Y Sun T Zheng P An J. Mass quarantine and mental health during COVID-19: a meta-analysis. J Affect Disord. (2021) 295:1335–46. doi: 10.1016/j.jad.2021.08.067, PMID: 34706447 PMC8674683

[2] Ornell F Schuch JB Sordi AO Kessler FHP. Pandemic fear and COVID-19: mental health burden and strategies. Braz J Psychiatry. (2020) 42:232–5. doi: 10.1590/1516-4446-2020-0008, PMID: 32267343 PMC7236170

[3] Brooks SK Webster RK Smith LE Woodland L Wessely S Greenberg N. The psychological impact of quarantine and how to reduce it: rapid review of the evidence. Lancet. (2020) 395:912–20. doi: 10.1016/S0140-6736(20)30460-8, PMID: 32112714 PMC7158942

[4] Wang C Pan R Wan X Tan Y Xu L Ho CS. Immediate psychological responses and associated factors during the initial stage of the 2019 coronavirus disease (COVID-19) epidemic among the general population in China. Int J Environ Res Public Health. (2020) 17:1729. doi: 10.3390/ijerph17051729, PMID: 32155789 PMC7084952

[5] Xiao H Zhang Y Kong D Li S Yang N. Social capital and sleep quality in individuals who self-isolated for 14 days during the coronavirus disease 2019 (COVID-19) outbreak in January 2020 in China. Med Sci Monit. (2020) 26:e923921–1. doi: 10.12659/MSM.923921, PMID: 32194290 PMC7111105

[6] Pierce M Hope H Ford T Hatch S Hotopf M John A. Mental health before and during the COVID-19 pandemic: a longitudinal probability sample survey of the UK population. Lancet Psychiatry. (2020) 7:883–92. doi: 10.1016/S2215-0366(20)30308-4, PMID: 32707037 PMC7373389

[7] Aqeel M Abbas J Shuja KH Rehna T Ziapour A Yousaf I. The influence of illness perception, anxiety and depression disorders on students’ mental health during COVID-19 outbreak in Pakistan: a web-based cross-sectional survey. Int. J Hum Rights Healthc. (2022a) 15:17–30. doi: 10.1108/IJHRH-10-2020-0095

[8] Auerbach RP Mortier P Bruffaerts R Alonso J Benjet C Cuijpers P. WHO world mental health surveys international college student project: prevalence and distribution of mental disorders. J Abnorm Psychol. (2018) 127:623. doi: 10.1037/abn0000362, PMID: 30211576 PMC6193834

[9] Ma Z Zhao J Li Y Chen D Wang T Zhang Z. Mental health problems and correlates among 746 217 college students during the coronavirus disease 2019 outbreak in China. Epidemiol Psychiatr Sci. (2020) 29:E181. doi: 10.1017/S2045796020000931 33185174 PMC7681173

[10] Pat-Horenczyk R Schiff M Arënliu A Zasiekina L Kagialis A Ferreira N. Challenges faced by university students during the COVID-19: An international study in five countries during the early phase of the pandemic. Int J Psychol. (2022) 57:547–58. doi: 10.1002/ijop.12846, PMID: 35567307 PMC9348485

[11] Alonzi S La Torre A Silverstein MW. The psychological impact of preexisting mental and physical health conditions during the COVID-19 pandemic. Psychol Trauma Theory Res Pract Policy. (2020) 12:S236–8. doi: 10.1037/tra0000840, PMID: 32525380

[12] Organización Internacional del Trabajo, (2020). Empleo juvenil en tiempos de la COVID-19: el riesgo de una generación del confinamiento Organización Internacional del Trabajo. Available at: https://www.ilo.org/wcmsp5/groups/public/−−-americas/−−-ro-lima/documents/briefingnote/wcms_753103.pdf

[13] Stroud I Gutman LM. Longitudinal changes in the mental health of UK young male and female adults during the COVID-19 pandemic. Psychiatry Res. (2021) 303:114074. doi: 10.1016/j.psychres.2021.114074, PMID: 34271372 PMC8520320

[14] Dosil-Santamaria M Ozamiz-Etxebarria N Idoiaga Mondragon N Reyes-Sosa H Santabárbara J. Emotional state of mexican university students in the COVID-19 pandemic. Int J Environ Res Public Health. (2022) 19:2155. doi: 10.3390/ijerph19042155, PMID: 35206340 PMC8871678

[15] Chávez IL. Ansiedad en universitarios durante la pandemia de COVID-19: un estudio cuantitativo. Psicumex. (2021) 11:1–26. doi: 10.36793/psicumex.v11i1.420

[16] Durán ME Rosales RA López CA. Cambios en los hábitos de sueño y el proceso educativo durante la jornada de sana distancia en estudiantes de una universidad pública: el caso de la Universidad Autónoma de la Ciudad de México, mayo 2020. Salud Problema. (2020) 14:36–56. Available at: https://repositorio.xoc.uam.mx/jspui/handle/123456789/25635

[17] Varjas K Nastasi BK Moore RB Jayasena A. Using ethnographic methods for development of culture-specific interventions. J Sch Psychol. (2005) 43:241–58. doi: 10.1016/j.jsp.2005.04.006, PMID: 22441174

[18] Figge CJ Martinez-Torteya C Taing S Chhim S Hinton DE. Key expressions of trauma-related distress in Cambodian children: a step toward culturally sensitive trauma assessment and intervention. Transcult Psychiatry. (2022) 59:492–505. doi: 10.1177/1363461520906008, PMID: 32178597

[19] Hinton DE Good BJ. Culture and PTSD: trauma in global and historical perspective. Philadelphia, PA: University of Pennsylvania Press (2016).

[20] Aqeel M Rehna T Shuja KH Abbas J. Comparison of Students’ mental wellbeing, anxiety, depression, and quality of life during COVID-19’s full and partial (smart) lockdowns: a follow-up study at a 5-month interval. Front Psych. (2022) 13:835585. doi: 10.3389/fpsyt.2022.835585, PMID: 35530024 PMC9067378

[21] Charbonnier E Le Vigouroux S Goncalves A. Psychological vulnerability of French university students during the COVID-19 pandemic: a four-wave longitudinal survey. Int J Environ Res Public Health. (2021) 18:9699. doi: 10.3390/ijerph18189699, PMID: 34574623 PMC8465825

[22] Censo de Población y Vivienda (2020). Monterrey Metro Área. Gobierno de México. Available at: https://datamexico.org/es/profile/geo/monterrey-991901

[23] López I. (2019). Ranking De Calidad De Vida. Mercer. Available at: https://www.latam.mercer.com/newsroom/estudio-calidad-de-vida.html

[24] Consejo Nacional de Evaluación de la Política de Desarrollo Social. (2020). Líneas de pobreza por ingreso. COVENAL InfoPobreza. Available at: http://sistemas.coneval.org.mx/InfoPobreza/Pages/wfrLineaBienestar?pAnioInicio=2016&pTipoIndicador=0

[25] de Salud Secretaria. (2020). Coronavirus (COVID-19) - Comunicados Técnicos Diarios. Gobierno de México. Available at: https://www.gob.mx/salud/documentos/coronavirus-covid-19-comunicados-tecnicos-diarios-septiembre-202028

[26] Corbin J Strauss A. Basics of Qualitative Research: Techniques and Procedures for Developing Grounded Theory. Thousand Oaks: SAGE Publications (2015).

[27] Applied Mental Health Research Group. (2013). The DIME program research model: design, implementation, monitoring, and evaluation. Johns Hopkins Bloomberg School of Public Health. Available at: https://www.jhsph.edu/research/centers-and-institutes/global-mental-health/resource-materials/design-implementation-monitoring-and-evaluation-dime/

[28] Yeh CJ Inman AG. Qualitative data analysis and interpretation in counseling psychology: strategies for best practices. Couns Psychol. (2007) 35:369–403. doi: 10.1177/0011000006292596

[29] Pérez-Aranda GI Estrada-Carmona S Catzin EA. Confinamiento y Ansiedad en estudiantes universitarios del sureste mexicano durante la pandemia de COVID-19. Comunidad y Salud. (2021) 19:25–32.

[30] Vital-López L García-García R Rodríguez-Reséndíz J Paredes-García WJ Zamora-Antuñano MA Oluyomi-Elufisan T. The impacts of COVID-19 on technological and polytechnic university teachers. Sustainability. (2022) 14:4593–620. doi: 10.3390/su14084593

[31] Audirac AM Zúñiga AGH Sosa JJS. Universitarios mexicanos: lo mejor y lo peor de la pandemia de COVID-19. Revista Digital Universitaria. (2021) 22. doi: 10.22201/cuaieed.16076079e.2021.22.3.12

[32] Bonanno GA Brewin CR Kaniasty K Greca AML. Weighing the costs of disaster: consequences, risks, and resilience in individuals, families, and communities. Psychol Sci Public Interest. (2010) 11:1–49. doi: 10.1177/1529100610387086, PMID: 26168411

[33] Robinson E Sutin AR Daly M Jones A. A systematic review and meta-analysis of longitudinal cohort studies comparing mental health before versus during the COVID-19 pandemic in 2020. J Affect Disord. (2022) 296:567–76. doi: 10.1016/j.jad.2021.09.098, PMID: 34600966 PMC8578001

[34] Kecojevic A Basch CH Sullivan M Davi NK. The impact of the COVID-19 epidemic on mental health of undergraduate students in New Jersey, cross-sectional study. PLoS One. (2020) 15:e0239696. doi: 10.1371/journal.pone.0239696, PMID: 32997683 PMC7526896

[35] Sánchez-Carlessi HH Yarlequé-Chocas LA Javier-Alva L Nuñez ER Arenas-Iparraguirre C Matalinares-Calvet ML. Anxiety, depression, somatization and experiential avoidance indicators in Peruvian university students in quarantine by covid-19. Revista de la Facultad De Medicina Humana. (2021) 21:346–53. doi: 10.25176/RFMH.v21i2.3654

[36] Gallagher S Sumner R Creaven AM O’Súilleabháin PS Howard S. Allostatic load and mental health during COVID-19: the moderating role of neuroticism. Brain Behav Immun-Health. (2021) 16:100311. doi: 10.1016/j.bbih.2021.100311, PMID: 34514440 PMC8419239

[37] Wilks CR Auerbach RP Alonso J Benjet C Bruffaerts R Cuijpers P. The importance of physical and mental health in explaining health-related academic role impairment among college students. J Psychiatr Res. (2020) 123:54–61. doi: 10.1016/j.jpsychires.2020.01.009, PMID: 32036074 PMC7047531

[38] Prout TA Zilcha-Mano S Aafjes-van Doorn K Békés V Christman-Cohen I Whistler K. Identifying predictors of psychological distress during COVID-19: a machine learning approach. Front Psychol. (2020) 11:586202. doi: 10.3389/fpsyg.2020.586202, PMID: 33240178 PMC7682196

[39] Hyphantis TN Tomenson B Bai M Tsianos E Mavreas V Creed F. Psychological distress, somatization, and defense mechanisms associated with quality of life in inflammatory bowel disease patients. Dig Dis Sci. (2010) 55:724–32. doi: 10.1007/s10620-009-0762-z, PMID: 19255844

[40] Galindo-Vázquez O Ramirez-Orozco M Costas-Muñiz R Mendoza-Contreras LA Calderillo-Ruíz G Meneses-García A. Síntomas de ansiedad, depresión y conductas de autocuidado durante la pandemia de COVID-19 en la población general. Gac Med Mex. (2020) 156:298–305. doi: 10.24875/GMM.20000266, PMID: 32831341 PMC8327400

[41] Priego-Parra BA Triana-Romero A Pinto-Gálvez SM Durán C Salas-Nolasco O Manriquez M. Anxiety, depression, attitudes, and internet addiction during the initial phase of the 2019 coronavirus disease (COVID-19) epidemic: a cross-sectional study in México. MedRxiv. (2020). doi: 10.1101/2020.05.10.20095844

[42] Reyes-Portillo JA Masia C Kline EA Bixter MT Chu BC Miranda R. The psychological, academic, and economic impact of COVID-19 on college students in the epicenter of the pandemic. Emerg Adulthood. (2022) 10:473–90. doi: 10.1177/21676968211066657, PMID: 38603124 PMC8832132

[43] Dhar BK Ayittey FK Sarkar SM. Impact of COVID-19 on psychology among the university students. Global Chall. (2020) 4:2000038. doi: 10.1002/gch2.202000038, PMID: 33042575 PMC7537036

[44] Jones HE Manze M Ngo V Lamberson P Freudenberg N. The impact of the COVID-19 pandemic on college students’ health and financial stability in new York City: findings from a population-based sample of City University of new York (CUNY) students. J Urban Health. (2021) 98:187–96. doi: 10.1007/s11524-020-00506-x, PMID: 33570739 PMC7877316

[45] Infante C Giraldo L Casas R. Informe de la Encuesta “Opiniones de los universitarios sobre el nuevo virus de influenza humana y sus efectos sociales”. Mexico City: Universidad Nacional Autónoma de México (2009).

[46] Estrada M. Convivencia forzosa: Experiencias familiares durante la emergencia sanitaria por el virus de la influenza humana A(HN1) en la Ciudad de México. Saberes y Razones Desacatos. (2020) 32:109–18.

